# Endometriosis-Associated Macrophages: Origin, Phenotype, and Function

**DOI:** 10.3389/fendo.2020.00007

**Published:** 2020-01-23

**Authors:** Chloe Hogg, Andrew W. Horne, Erin Greaves

**Affiliations:** ^1^Medical Research Council Centre for Reproductive Health, The University of Edinburgh, Edinburgh, United Kingdom; ^2^Division of Biomedical Sciences, Warwick Medical School, University of Warwick, Coventry, United Kingdom

**Keywords:** endometriosis, macrophage, monocyte, origin, phenotype

## Abstract

Endometriosis is a complex, heterogeneous, chronic inflammatory condition impacting ~176 million women worldwide. It is associated with chronic pelvic pain, infertility, and fatigue, and has a substantial impact on health-related quality of life. Endometriosis is defined by the growth of endometrial-like tissue outside the uterus, typically on the lining of the pelvic cavity and ovaries (known as “lesions”). Macrophages are complex cells at the center of this enigmatic condition; they are critical for the growth, development, vascularization, and innervation of lesions as well as generation of pain symptoms. In health, tissue-resident macrophages are seeded during early embryonic life are vital for development and homeostasis of tissues. In the adult, under inflammatory challenge, monocytes are recruited from the blood and differentiate into macrophages in tissues where they fulfill functions, such as fighting infection and repairing wounds. The interplay between tissue-resident and recruited macrophages is now at the forefront of macrophage research due to their differential roles in inflammatory disorders. In some cancers, tumor-associated macrophages (TAMs) are comprised of tissue-resident macrophages and recruited inflammatory monocytes that differentiate into macrophages within the tumor. These macrophages of different origins play differential roles in disease progression. Herein, we review the complexities of macrophage dynamics in health and disease and explore the paradigm that under disease-modified conditions, macrophages that normally maintain homeostasis become modified such that they promote disease. We also interrogate the evidence to support the existence of multiple phenotypic populations and origins of macrophages in endometriosis and how this could be exploited for therapy.

## Background

Endometriosis is defined by the presence of endometrial-like tissue outside the uterus (“lesions”), typically on the lining of the pelvic cavity (peritoneum) or on the ovaries. Endometriosis is a heterogeneous disease, and lesions can be categorized into three sub-types: superficial peritoneal, deep (infiltrating), and ovarian (“endometriomas”), where more than one sub-type can exist in the same patient and superficial peritoneal endometriosis is the most common form of disease (1, 2). It is associated with debilitating chronic pelvic pain, infertility, and fatigue. It is estimated to affect 6–10% of women of reproductive age (3), up to 50% of infertile women (4) and is prevalent in 71–97% of women with chronic pelvic pain (5). Endometriosis-associated symptoms can negatively impact mental, physical and social well-being and quality of life (6). Poor pregnancy outcomes are also associated with the disease, including preterm labor, pre-eclampsia, ectopic pregnancy, miscarriage, and intrauterine growth restriction (7). Endometriosis has a significant socioeconomic impact, costing the UK an estimated £8.5 billion pounds each year, with societal cost being mostly attributed to loss of productivity (8, 9). Diagnosis from onset of symptoms can take an average of 7–8 years. Generally, a diagnosis of endometriosis is achieved by laparoscopic evaluation of the pelvis, however imaging techniques such as transvaginal sonography and magnetic resonance imaging may be utilized to diagnose deep lesions and endometriomas (10–12).

**Figure 1:**
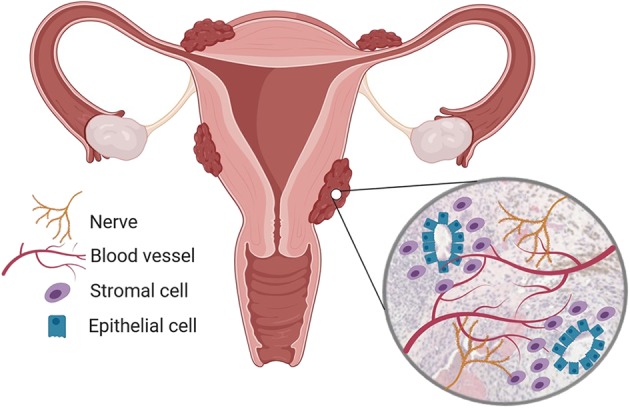
Endometriosis is a chronic inflammatory condition. Endometriosis is characterized by the presence of endometrial-like tissue found outside the uterus, most commonly in the peritoneal cavity. Endometriosis lesions are heterogenous but usually contain endometrial stromal cells and epithelial glands, immune cell infiltrates and are vascularized and innervated by nerves. Created using Biorender.com.

Endometriosis lesions are characterized by the presence of ectopic endometrial-like tissue containing glands and stroma, however recent re-evaluation of disease definition suggests that fibrosis and smooth muscle cells are more consistent features of lesions (13). Endometriosis is classified as an estrogen-dependent *chronic inflammatory condition*: symptoms are modulated by ovarian hormones and lesions generate intense inflammation within the pelvic cavity. Lesions also become vascularized and are infiltrated by sensory nerve fibers (Figure 1). The ectopic endometrial cells and local inflammatory environment activate nerve fibers in lesions, establishing a dialogue with the central nervous system and generating pain in the condition. Lesions behave like the eutopic endometrium and exhibit cyclical bleeding into the pelvic cavity in response to ovarian hormones, and this acts to potentiate inflammation (14). Disease classification (rAFS/rASRM) is currently based on lesion size, location, extent of lesion infiltration into tissue and the presence of adhesions. Classification ranges from stage I (“minimal”) to stage IV (“severe”) (15).

Current treatments for endometriosis aim to alleviate endometriosis-associated pain and/or to treat infertility associated with the disease and include surgical and medical management (2, 3). Ovarian suppression limits activity and growth of lesions, leading to reduced pain symptoms. Common methods of ovarian suppression include oral contraceptives and gonadotrophin-releasing hormone (GnRH) agonists (16) with add-back HRT. Whilst ovarian suppression may alleviate pain symptoms, treatment is also contraceptive and therefore inappropriate for women aiming to conceive. Additionally, GnRH agonists are associated with side effects such as memory loss, insomnia, and hot flushes in a recent study of endometriosis patients with long term use (17). Treatments can also include non-steroidal anti-inflammatory drugs such as ibuprofen, however long-term pain management for women with endometriosis often encompasses a combination of treatments. As well as medical therapy, laparoscopic surgery to remove lesions can provide symptom relief in some patients, however up to 50% of women experience a relapse of symptoms within 2 years after surgery (11). Current treatment options lack significant clinically proven benefit and aim at alleviating symptoms, rather than treating disease (18). Consequently, there is a compelling clinical need for new non-hormonal treatments that have fewer side effects and effectively treat endometriosis over a life course, without the need for repeated surgeries or suppression of fertility.

## Etiology and Natural History

It is widely accepted that endometriosis is a multifactorial disease and the pathophysiology of endometriosis can certainly be associated with a number of elements that clearly contribute to disease. Evidence suggests that endometriosis has a heritable component due to high familial incidence of the disease (19–22). A meta-analysis of eight genome-wide association studies (GWAS) elucidated six loci associated with endometriosis (23). Genes implicated in disease included those involved in the regulation of epithelial cells and hormone metabolism, specifically genes involved in regulating hormone responses in tissues (24, 25). These GWAS results are not surprising since the symptoms of endometriosis are modulated by ovarian sex steroids; early age at menarche is a risk factor for development of endometriosis, suggesting increased exposure to estrogen may incur increased risk of disease (26). Endometriosis lesions aberrantly express a number of steroidogenic enzymes including aromatase and 17β-hydroxysteriod dehydrogenase (17β-HSD), this results in increased synthesis and decreased metabolism of estrogen (27–29) such that local levels remain high. Estrogen signaling modulates a large number of down-stream disease processes within endometriosis lesions, which are reviewed in Yilmaz and Bulun (30), Liang et al. (31), and Rizner (32). Immune cell dysfunction is also intrinsically linked to the pathophysiology of endometriosis. Alterations in immune cell populations have been observed in the peritoneal fluid of women with endometriosis; specifically, women with endometriosis have more peritoneal macrophages (33), neutrophils and dendritic cells (34). Function is also perturbed: NK cells have reduced cytotoxicity (35, 36), and disease severity is positively correlated with NK cell killing capacity (37). Peritoneal macrophages also exhibit impaired phagocytosis (38). Macrophages are the most abundant immune cells present within endometriosis lesions and are evidently central to the pathophysiology of endometriosis. Whilst studies have highlighted clear functional roles for macrophages in the disorder, little is known regarding the origins and phenotypic heterogeneity of macrophages in endometriosis.

Our understanding of endometriosis etiology remains limited. It is being increasingly recognized that different sub-types of endometriosis may arise from different origins, however evidence for this is still limited (39, 40). A number of theories are discussed below and we speculate on how the origin and role of macrophages may differ in each scenario:

The most widely accepted theory was postulated in 1927 by John Sampson, who observed that during menstruation, endometrial tissue can reflux back up the fallopian tubes and into the pelvic cavity, a physiological process known as “*retrograde menstruation*.” Although this process occurs in ~90% of women, only in some does refluxed endometrial tissue form endometriosis lesions (41) and the mechanisms underpinning the attachment of endometrial tissue and lesion development remain elusive. It could be predicted, and mouse studies have demonstrated that macrophages originating from the endometrium contribute to peritoneal endometriosis lesions (42). These endometrial macrophages could be pivotal in the establishment of lesions since it has previously been demonstrated that macrophages trafficking to the endometrium are most abundant during repair following endometrial breakdown and shedding with a presumed role in repairing the denuded functional layer of the endometrium (43). However, evidence supporting this hypothesis is still absent. Another theory based on the dissemination of cells from the uterus into the peritoneal cavity suggests that neonatal retrograde reflux of endometrial stem/progenitor cells could be responsible for development of lesions. Visible vaginal bleeding is observed in 3–5% of female neonates, whereas occult bleeding may occur at a frequency of between 25 and 60% (44). Bleeding in the immediate postnatal period is similar to menstrual bleeding as it occurs in response to hormone withdrawal from *in utero* progesterone exposure. This theory suggests that stem/progenitor cells could implant into the peritoneal wall where they may remain dormant until adolescence, when elevated estrogen levels may then promote the proliferation and growth of seeded endometrial cells. Whilst, this theory represents a plausible mechanism of lesion formation, current evidence is lacking and proof that endometrial stem/progenitor cells are present in the peritoneal tissue of pre-pubescent girls is absent. The *coelomic metaplasia* theory suggests that endometriosis lesions arise as the result of metaplastic differentiation of the coelomic epithelium into endometrial cells and is supported by evidence suggesting endometriosis lesions can be found in women without a uterus (45). The formation of endometriosis lesions at sites distant from the peritoneal cavity (46, 47), as well as identification in men on rare occasions (48) supports the theory. Upon development of lesions at the onset on adolescence (neonatal stem cell theory) or following metaplasia it would be expected that monocytes are recruited to the site of the lesion and/or that peritoneal macrophages may traffic into the developing lesion and activate repair processes that facilitate establishment of new endometrial-like explants. Notably, stem cells and macrophages are known to have a reciprocal relationship whereby stem cells can contribute to macrophage activation and phenotype during regenerative processes and macrophages can dictate accumulation of progenitor/stem cell-like cells (49). In endometriosis, mesenchymal stem-like cells promote macrophages to adopt a pro-repair phenotype (50) but further studies regarding the relationship between stem cells and macrophages in endometriosis are currently limited. *Müllerianosis* (müllerian rests; normal endometrial, endosalpingeal, and endocervical tissue) predicts that developmentally displaced tissue are incorporated into normal organs during organogenesis (51). Occurrence of deep infiltrating endometriosis particularly lends itself to this theory, where endometrial tissue is found deep within the organ structure. Speculation may infer a role for tissue-resident macrophages in lesions resulting from developmentally displaced endometrial-like tissue. Upon activation of a “dormant” lesion laid down during organogenesis the tissue-resident macrophages may change phenotype and proliferate such that they promote inflammation, growth, and invasion of the lesion. Inflammation arising upon activation of a dormant lesion may also lead to the recruitment of monocytes that differentiate into macrophages such that endometriosis lesion-resident macrophages are constituted by tissue-resident and monocyte-derived macrophages similar to what occurs in tumors (52). Any differences existing in macrophage origin, phenotype and function across the different subtypes of endometriosis lesions remain unknown.

## The Macrophage: a Complex Cell at the Center of an Enigmatic Condition

Inflammation and immune cell dysfunction are central to the pathophysiology of endometriosis. Whilst, a number of leukocytes exhibit altered numbers and function in endometriosis, it is evident that macrophages play an unrivaled role in disease pathogenesis. We and others have demonstrated that macrophages are critical for licensing lesion growth, promoting vascularization and innervation as well as contributing to pain in the disorder (53–55). Lessons from diverse tissues also place macrophages at the center of disease states such as liver injury (56), multiple sclerosis (57), and cancer (52). Tissue context ultimately dictates the role that macrophages play in disease but a recurring theme indicates that the ontogeny of the macrophages in diseased tissues determines how they respond and contribute to pathogenesis. Below, we review the available literature on macrophage ontogeny, phenotype and function in health and then focus on their role during inflammation and disease states. Ultimately, we discuss the role that macrophages play in endometriosis in light of what can be learnt from other disease states.

### Macrophages Have Different Origins and Diverse Phenotypes

#### Macrophage Ontogeny

**Figure 2:**
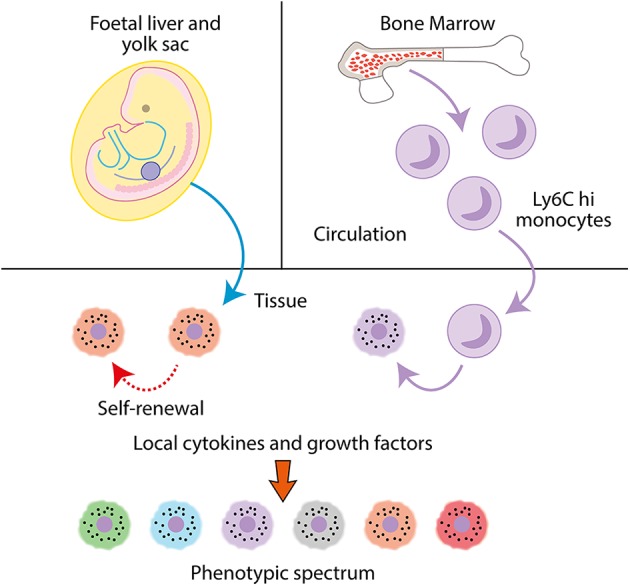
Macrophages are mononuclear phagocytes. Tissue macrophages are seeded during fetal life from the fetal liver and yolk sack and undergo self-renewal. In adults, monocyte precursors extravasate from the bone marrow into the circulation, where they can then infiltrate into tissues and differentiate into macrophages. In tissues, macrophages modulate their phenotype dependent on local cytokines and growth factors to specific tissue or disease-associated phenotypes.

Macrophages are mononuclear phagocytes that play critical roles in immunity (phagocytizing pathogens, apoptotic cells and debris, antigen presentation, and modulation of other leukocyte populations). They are present in all tissues of the body (58, 59) and play diverse tissue specific roles in maintaining homeostasis. Much of our knowledge regarding macrophage ontogeny is derived from studies conducted in mice. Macrophages are derived from three key populations; the yolk sac of the embryo, the fetal liver and postnatally, hematopoiesis in the bone marrow (Figure 2). The earliest macrophages arise from erythro-myeloid progenitors (EMPs) produced during primitive hematopoiesis in the extra-embryonic yolk sac at embryonic day (E) 7.5 and 8.25. After blood circulation is established, EMP derived macrophages seed fetal tissue. Excluding microglia, these macrophages are partially or fully replaced by monocytes originating from the fetal liver, which differentiate into macrophages in tissues. Fetal liver monocyte-derived macrophages can persist into adulthood and form the tissue resident macrophage population, undergoing self-renewal, for example in the peritoneum, spleen, lung, skin, and liver. In other organs, tissue macrophages derived from fetal liver monocytes are gradually replaced by recruited monocytes from the bone marrow. This process occurs in tissues such as the gut and dermis (60–64).

In humans, peripheral blood monocytes form two main populations; CD14^hi^ CD16^lo^ and CD14^lo^ CD16^hi^, although an intermediate population can be identified. The CD14^hi^ CD16^lo^ (classical) subset is the most abundant in the blood. Under inflammatory conditions, classical monocytes extravasate into tissues, differentiate into macrophages or dendritic cells (65) and fulfill functions such as clearance of apoptotic bodies, stimulating angiogenesis and restoring integrity of tissues (66). CD14^lo^ CD16^hi^ (“non-classical”) monocytes also exhibit extravasation into tissues during inflammation, but they infiltrate tissues later in the inflammatory process and exhibit a bias toward differentiating into “wound-healing” macrophages (67). A key role of the non-classical monocyte population is to patrol the blood vessels along the endothelial cell layer, providing immunosurveillance of vasculature and the surrounding tissues (68). Classical monocytes also patrol tissues and play a homeostatic role in steady state conditions, without differentiating into macrophages (69). Classical and non-classical monocytes in humans are analogous to Ly6C^hi^ classical and Ly6C^lo^ non-classical monocytes in mice and exhibit significant homology at transcriptional analysis (70, 71). In mice, classical monocytes can be classified as Ly6C^hi^ CX3CR1^lo^ CD43^lo^CCR2^hi^, and non-classical monocytes as Ly6C^lo^ CX3CR1^hi^ CD43^hi^CCR2^lo^, with all monocyte populations being CD11b^hi^ F4/80^int^ (65).

#### Macrophage Phenotype

Macrophages respond to their local microenvironment and change both their transcriptome and phenotype in response to local signals (72). Historically, macrophages have been divided into either “M1” classically or “M2” alternatively activated cells. Classically activated macrophages are associated with inflammation, and express pro-inflammatory markers. Alternatively activated macrophages are associated with homeostasis, wound healing and immunomodulation (73). These extreme polarization states only really exist *in vitro*, where studies commonly use stimulation with granulocyte-macrophage colony-stimulating factor (GM-CSF) and the cytokine IFN-γ (Interferon γ) to generate M1 macrophages, and stimulation with macrophage colony-stimulating factor (M-CSF) and the cytokines interleukin-4 (IL-4) and interleukin-10 (IL-10) to generate M2 activated macrophages (74). Whilst, this classification system is a useful tool for investigating macrophages at extremes of activation, it is now appreciated that *in vivo* macrophages exhibit a broad spectrum of phenotypes that are tissue and disease specific, and the M1/M2 system cannot represent the diverse nature and complexities of macrophage phenotype (75, 76). Transcriptional analysis of mouse macrophage populations from different tissues demonstrates minimal overlap in mRNA expression, reflecting a divergence in gene expression patterns (77). This heterogeneity reflects the ability of macrophages to modulate their gene expression in response to local tissue signals, becoming specialized to their tissue niche, be that in a healthy or diseased state. In disease, macrophages may modulate their phenotype dependent on disease stage or severity, and the mechanisms behind this are crucial for understanding their exact role in pathogenesis. Thus, defining macrophage phenotypes in disease states, with the potential of modulating macrophage phenotype or specifically targeting disease specific macrophages for clinical benefit is a key focus for research (72, 78–80).

### Endometrial Macrophages

The endometrium is a unique and highly dynamic tissue that undergoes cyclic proliferation, differentiation, shedding (menstruation), and repair in response to ovarian-derived estrogen and progesterone during the menstrual cycle. In the normal cycling endometrium, an influx of macrophages occurs during the secretory and menstrual phases, along with a concomitant increase in macrophage-derived cytokines and proteases (81). Evidence from a mouse model of endometrial breakdown and repair identified an influx of classical monocytes which differentiated into macrophages in the endometrium during the repair phase of the menstrual cycle (43). Monocyte extravasation from blood vessels into the endometrium is regulated by CCL2 (82, 83) and CX3C chemokine receptor 1 (CX3CR1) (84). The influx of macrophages into the endometrium is in line with the numerous roles they are presumed to play in modulating endometrial differentiation, breakdown, and repair. During the proliferative phase, macrophages have been postulated to play a role in regeneration and proliferation of the functional layer of the endometrium and express activation and adhesion markers CD54, CD69, and CD71 (85). Macrophages are also implicated in regulating gland remodeling (86) and angiogenesis during the secretory phase via production of vascular endothelial growth factor (VEGF) (87). At menstruation macrophages play a role in initiation of endometrial shedding by secreting matrix metalloproteinases (MMPs) (88). Specifically, secretion of MMP-12, MMP-9, and MMP-14 are required for the breakdown of the functional layer of the endometrium during menstruation (89–91).

In response to estrogen, macrophages increase their proliferative capacity and undergo activation to adopt a phenotype which represents a more “wound healing-like” population (92). Thus, estrogen signaling can accelerate the wound healing process and this is in part regulated by increasing the production of macrophage-derived proteases, MMPs, fibroblast growth factor, VEGF and cytokines such as resistin like alpha (RELMα) (92–94). Endometrial macrophages do not express the progesterone receptor (95), however macrophage gene expression is significantly altered in response to progesterone (96) suggesting an indirect method of regulation. Interestingly, exposure to cortisol was demonstrated to increase expression of angiogenic genes such as CXCL2, CXCL8, and VEGFC in macrophages *in vitro*, suggesting that local cortisol levels could be important for regulating angiogenesis within the remodeling endometrium (97). Taken together this evidence indicates that macrophages are key players in augmenting dynamic remodeling and repair in the endometrium and this is regulated by exposure to local cytokines, growth factors and hormones that modulate their phenotype, function, and recruitment throughout the menstrual cycle. However, compared to other tissue macrophages, the phenotype and function of endometrial macrophages and the mechanisms governing their recruitment and activation are significantly less well-characterized.

### Peritoneal Macrophages

#### Mouse

Peritoneal cavity macrophages are one of the most studied macrophage populations in mice, largely due to their ease of isolation. Two subsets of peritoneal macrophages are recognized in mice based on differential expression of F4/80 and MHC II. The tissue resident, so called “large” (due to their larger size) peritoneal macrophages (LpM) are F4/80^hi^, MHC II^lo^, and the monocyte-derived “small” peritoneal macrophages (SpM) are F4/80^lo^ MHC II^hi^ (98). LpM are the most abundant macrophage population in the peritoneal cavity at steady state and form the tissue resident population, they are phagocytic and perform immunosurveillance and homeostatic roles in the peritoneal cavity (98) as well as mediating recruitment and maintenance of B1 B cells. They are also linked to regulation of intestinal immunity (99). LpM self-renew and the proliferative capacity of LpM is regulated by GATA-binding factor 6 (Gata6), a transcription factor uniquely expressed by LpM in the peritoneal cavity, which also regulates macrophage phenotype (100). In mice, the LpM population consists primarily of embryonic-derived cells, however monocyte-derived macrophages do replace embryonic-derived LpM over time, a process that is highly sex and age dependent, and slower in females. Over time, Ly6C^hi^ monocytes enter the peritoneal cavity in a CCR2-dependent manner and differentiate transiently into SpM, prior to transitioning into tissue resident LpM (101). Thus, the LpM constitute both embryonic and monocyte-derived cells and the two populations have been shown to be transcriptionally distinct from each other (101). SpM are implicated in the inflammatory response in the peritoneal cavity, however their role in the steady state peritoneal cavity remains unclear (102).

#### Human

In humans, macrophages constitute 50% of peritoneal cavity leukocytes (103). Tissue resident peritoneal macrophages have been defined by high expression of complement receptor of the immunoglobulin superfamily (CRIg) and low expression of CCR2. These cells are highly phagocytic and more numerous in steady state, also displaying similar transcriptional profiles to the mouse LpM population (104). Human monocyte-derived macrophages in the peritoneal cavity, analogous to F4/80^lo^ MHC II^hi^ SpM in the mouse, have been defined as CRIg^lo^, CCR2^hi^. This CRIg^lo^, CCR2^hi^ population in humans has a reduced phagocytic capacity and is lower in number compared to CRIg^hi^ CCR2^lo^ tissue macrophages, consistent with characteristics of SpM. It must be noted however that in humans, Gata6 was found to be more highly up-regulated in the pro-inflammatory CRIg^lo^ CCR2^hi^ population (104), highlighting that key differences between human and mouse peritoneal macrophages exist, and further research is critically required to clarify these differences.

#### Peritoneal Macrophage Dynamics During Inflammation

Under inflammatory conditions, LpM respond to stimuli in a phenomenon known as the macrophage disappearance reaction (MDR) (105): in mice the LpM compartment undergoes a dramatic reduction in numbers largely by migration to the omentum, mediated by retinoic acid and Gata6 (106). The degree of loss in the LpM population is highly dependent on the dose of inflammatory stimuli and has been studied in a number of inflammatory models, such as lipopolysaccharide (LPS), zymosan or thioglycollate induced peritonitis (107–109). LpM that persist during inflammation have been hypothesized to play a regulatory role in the peritoneal cavity by secretion of IL-10, an anti-inflammatory cytokine which has also been shown to regulate inflammatory SpM number (109). LpM also play a key role in clearance of apoptotic cells during inflammation (108), and exhibit high expression of T-cell immunoglobulin and mucin domain containing 4 (Tim4) which recognizes phosphatidyl-serine on apoptotic cell bodies (110). Upon resolution of inflammation, the depleted LpM population increases its proliferative capacity through a colony stimulating factor 1 receptor (Csf-1r) mediated mechanism to restore LpM number (107). Interestingly, LpM have been shown to infiltrate the liver by a non-vascular route in response to the damage-associated molecular pattern molecule (DAMP) ATP, where they play a key role in regeneration and tissue repair in the liver after sterile injury, modulating their phenotype in response to local tissue microenvironmental cues (111). This migration implies that LpM have the ability to execute wound repair and tissue regeneration in visceral organs. Furthermore, with a reduction of LpM numbers a concurrent increase in SpM and inflammatory Ly6C^hi^ monocytes is observed in a number of mouse models of peritoneal inflammation (105). SpM exhibit a pro-inflammatory response when challenged with LPS *in vitro*, producing high levels of chemokine (C-C motif) ligand 5 (Ccl5), chemokine (C-C motif) ligand 3 (Ccl3), and tumor necrosis factor-α (Tnf-α), as opposed to LpM which produce G-CSF and GM-CSF under LPS stimuli (102). In an *in vivo* model of peritonitis, SpM also produce high amounts of pro-inflammatory cytokines including Tnf-α, interleukin-1β (Il-1β), and Ifn-γ (112), and are critical for clearance of infection in the peritoneal cavity after bacterial challenge in the mouse (113). The ability of SpM to respond to inflammatory stimuli by producing pro-inflammatory cytokines enables rapid response to immunological challenge in the peritoneal cavity. At resolution of inflammation, SpM have been shown to undergo apoptosis (108) but can also migrate to local draining lymph nodes (114). However, SpM have also been shown to persist in the cavity and can eventually differentiate into F4/80^hi^ MHC II^lo^ cells (115), suggesting that inflammation has the potential to alter the complement of peritoneal cavity macrophage populations, even after homeostasis has been restored. The multiple fates of SpM reflect the heterogeneity in this cell compartment, but the roles of SpM sub-populations in inflammation are still largely undefined. In summary, under steady-state/homeostatic conditions LpM exhibit an immune-surveillance and immune-regulatory role and act to remove apoptotic and senescent cells. The roles of SpM are less well-defined but the markers they express suggests roles in antigen presentation and T cell activation. Inflammatory challenge with thioglycolate, zymosan or LPS (lipopolysaccharide) causes loss of LpM and expansion of SpM via monocyte recruitment and differentiation. New SpM are pro-inflammatory, expressing high levels of Tnfα, Il-1β, and Ifnγ and are better able to engulf microbes compared to homeostatic SpM. Of note, type-2 inflammation characterized by elevated levels of IL-4 does not induce MDR and instead F4/80^hi^ LpM accumulate in the peritoneal cavity and exhibit a pro-repair phenotype (116). Thus, it seems that under different inflammatory challenge LpM are biased to exhibit a pro-repair phenotype whilst SpM adopt a pro-inflammatory phenotype. Mechanistic studies on peritoneal macrophages in humans are challenging and therefore knowledge of this physiological process in humans is minimal.

### Macrophages Can Promote Disease

The unique and diverse roles that macrophages play in the maintenance of healthy tissues is mirrored by their pivotal roles in development, maintenance, and progression of a number of diseases (72). Peritoneal cavity macrophage perturbations and functional dysregulation are linked to a number of adverse clinical outcomes. For example, an increase in peritoneal macrophages was associated with negative outcomes in patients with peritonitis (109), and dysregulation of peritoneal macrophages has been linked to acute pancreatitis, where peritoneal macrophages produce increased levels of pro-inflammatory cytokines that exacerbate disease (115). Conversely, macrophages have been shown to be protective against the formation of adhesions, a common complication after abdominal surgery (117) and indicating that macrophage dysfunction could contribute to adhesion formation. Thus, macrophages are intrinsically linked to disease in the peritoneal cavity in humans.

Although endometriosis is a benign condition a number of parallels can be drawn between the condition and cancer (118). Macrophages are unambiguously at the center of the pathophysiology of both diseases. Macrophage infiltration in tumors is a predictor of poor clinical outcomes in malignancy (119, 120), attributed to the fact that macrophages promote initiation, progression, and metastasis in most cancers (75). In the last decade, a major focus has been to define the populations that constitute tumor-associated macrophages (TAMs). In a mouse model of breast cancer, Ly6C^hi^ inflammatory monocytes are recruited to metastatic sites via a CCR2/CCL2 mediated mechanism to form TAMs. Inhibition of CCL2/CCR2 signaling with an anti-CCL2 antibody inhibited monocyte recruitment thereby inhibiting metastasis and prolonging survival of the mice (121). Similarly, mouse models of Lewis lung carcinoma demonstrated that TAMs were derived from CCR2 driven recruitment of Ly6C^hi^ monocytes and blockage of CCL2 decreased tumor growth (122, 123). Furthermore, tissue resident macrophages have also been implicated in cancer pathophysiology and can contribute to the TAM population. For example, in a mouse model of pancreatic ductal adenocarcinoma, Zhu et al. demonstrated using a parabiosis model that TAMs were derived from both embryonically derived tissue resident macrophages as well as from circulating Ly6C^hi^ monocytes. During tumor development embryonically derived macrophages expanded via *in situ* proliferation and had a pro-fibrotic role in tumors. Using a Csf-1r neutralizing antibody and clodronate liposome treatment to deplete tissue resident macrophages a reduction in tumor size and increased survival of mice was observed. Monocyte-derived macrophages however played a key role in antigen presentation. Use of CCR2 knockout mice or a CCR2 inhibitor to prevent recruitment of Ly6C^hi^ monocytes did not affect tumor growth (52). This study highlights the importance of defining the ontogeny of TAMs in order to decipher which populations are fundamentally required for tumor growth, with the aim of improving clinical outcomes.

Whilst TAMs may have multiple origins, it has been demonstrated that the tumor microenvironment can modulate macrophage phenotype to promote malignancy, indicating that origin does not wholly define function when macrophages are exposed to cytokines and growth factors locally in the tumor. A number of different macrophage populations within tumors have been described which play differential roles and have different phenotypes. For example, populations of invasive, perivascular, metastasis associated, angiogenic (Tie2^+^), and immunosuppressive macrophages which secrete high levels of IL-10 have been described (75). Detailed profiling of hepatocellular carcinoma biopsies demonstrated the presence of various macrophage sub-types in tumors that had both pro and anti-tumoral properties (124).

### The Role of Macrophages in Endometriosis

#### Macrophage Ontogeny in Endometriosis

**Figure 3:**
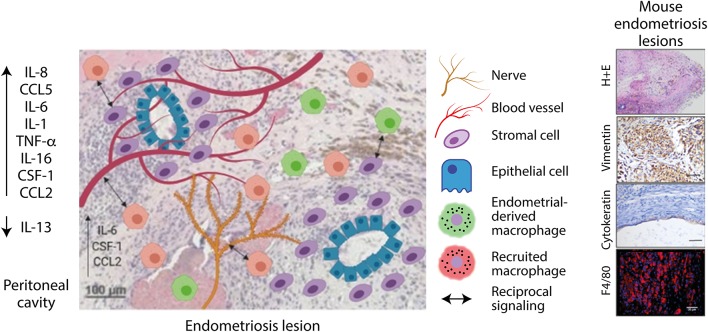
Endometriosis lesions are infiltrated by blood vessels, nerves, and macrophages. Lesion resident macrophages are derived from macrophages originating from the endometrium and recruited macrophages. Macrophages interact with blood vessels and nerves to stimulate their growth. Signaling also occurs between macrophages and stromal cells, which increases their clonal expansion and invasive properties. Created using Biorender.com.

Whilst, a role for macrophages in endometriosis pathophysiology is established (and discussed below), the ontogeny of endometriosis-associated macrophages is still poorly understood. Greaves et al. demonstrated in a syngeneic mouse model of endometriosis that lesion resident macrophages are derived from both the (donor) endometrium and (recipient) infiltrating macrophage populations (42) (Figure 3). These infiltrating macrophage populations are likely to constitute peritoneal or recruited monocyte-derived macrophages, however the exact origins of these populations is currently unknown. Although, peritoneal macrophages contribute to inflammation in endometriosis, it remains unknown whether they infiltrate endometriosis lesions and thus the role these cells play within the ectopic tissue is not known. Using bone marrow chimeras Sekiguchi et al. demonstrated that CD11b^+^ cells from the bone marrow infiltrate and accumulate in endometriosis lesions in a mouse model (125). These cells could represent a monocyte/macrophage population, although CD11b^+^ cells could also constitute neutrophils, eosinophils and or certain subsets of dendritic cells (126). Capobianco et al. demonstrated that bone marrow derived Tie2^+^ cells infiltrated endometriosis lesions in a mouse model, again demonstrating that bone marrow derived cells that ultimately express macrophage markers within lesions could be recruited from blood vessels (127).

#### Macrophage Phenotype and Function in Endometriosis

Endometrial macrophages exhibit differential properties in endometriosis. Reflecting on the theory of retrograde menstruation and studies in mice identifying endometrial macrophages in lesions, the presence of macrophages in refluxed endometrial tissue in women has the potential to augment disease development in the peritoneal cavity. A number of studies have demonstrated perturbations in macrophage populations in the eutopic endometrium of endometriosis patients. Women with endometriosis have more endometrial macrophages that express lower levels of the “wound-healing” marker CD163 compared to women without disease, however the exact mechanisms behind these alterations are unknown (128, 129). Analogous to this, increased levels of CCL2 can be observed in the endometrium of women with disease which corresponds to disease severity, suggesting increased influx of monocytes in disease that can then differentiate into macrophages (130). Increased matrix metalloproteinase-9 (MMP-9) co-localized with CD68^+^ macrophages in the endometrium of women with endometriosis is indicative of an increase in the number of macrophages implicated in tissue remodeling. This may enhance the ability of ectopic endometrial tissue deposits to implant in the peritoneal cavity (131). Whilst evidence of macrophage perturbations in the eutopic endometrium of women with endometriosis exists, the role of endometrial macrophages in endometriosis has not been defined as functional studies in this area are lacking.

Women with endometriosis evidently have an increased number of peritoneal macrophages that exhibit a dysfunctional phenotype. Peritoneal macrophages collected from women with endometriosis have reduced phagocytic capacity due to low levels and activity of matrix metalloproteinase 9, which is required for extracellular matrix degradation and is regulated by prostaglandin E2 (PGE2) (38). In a co-culture system, in the presence of endometrial stromal cells isolated from ectopic endometrial tissues, monocyte-derived macrophages secreted IL-10 and TGF-β, which in turn suppressed cytotoxicity and viability of NK cells (132), suggesting that macrophages are immunosuppressive in the presence of ectopic endometrial stromal cells and can act to suppress NK cells in the peritoneal cavity. Whilst, a few studies have investigated peritoneal macrophages in women with endometriosis the cells have been evaluated as a global population and there are no studies pertaining to the constitution and function of the individual CRIg^hi^ and CRIg^lo^ populations of these cells: the abundance and behavior of CRIg^hi^ population in women with endometriosis is not known, although inflammation and survival of refluxed endometrial tissue suggests that in endometriosis this tissue resident population could act to create a permissive and mitogenic environment for the formation of lesions. Analogous to this, a study by Beste et al. demonstrated enhanced expression of both pro- and anti-inflammatory cytokines by macrophages collected from the peritoneal fluid of women with endometriosis, this could reflect the mixed population of cells present (133). The increased number of peritoneal macrophages in women with endometriosis suggests that in the condition the “macrophage disappearance reaction” (MDR) does not occur. Indeed, it has been previously demonstrated that in type-2 inflammation characterized by high level of IL-4 the MDR does not happen and peritoneal macrophages accumulate as a result of *in situ* proliferation (116). IL-4 concentrations are elevated in the peritoneal fluid of women with endometriosis (134) suggesting that this could be a mechanism for macrophage accumulation. However, because the abundance of the different populations has not been characterized this hypothesis remains to be proven. Mouse models of endometriosis provide conflicting evidence of peritoneal macrophage dynamics: Yuan et al. demonstrated that in a model that injects syngeneic, estradiol primed, endometrial fragments into intact mice, those with endometriosis exhibited significantly lower numbers of LpM and more abundant SpM compared to control mice, consistent with the MDR. These perturbations in peritoneal macrophage populations were evident from 0.25 to 42 days post tissue injection (135). However, in a model injecting “menses-like” endometrial tissue into ovariectomized recipients supplemented with estradiol valerate, loss of LpM was not observed, and mice with endometriosis had more abundant LpM compared to naïve and sham animals [although the increase was not statistically significant (54)]. The second study seems to more closely recapitulate macrophage dynamics in women with endometriosis, although evidence is very limited. The differences observed in these two studies could be a result of several differences in experimental design including the nature of the donor endometrium injected into the peritoneal cavity as well as manipulations performed on the recipient mice. Yuan et al. also demonstrated that in mice with endometriosis LpM exhibited a “pro-inflammatory” activation state and SpM were more “pro-repair” in nature (135). This interpretation was based on expression of NOS2 (inflammatory) and CD206 (repair) and is contradictory to others studies reporting the pro-inflammatory status of SpM and pro-repair status of LpM in response to different inflammatory stimuli.

Although, it has been demonstrated that monocytes are recruited to lesions from the bone marrow, little evidence exists to characterize their role and dynamics once they infiltrate ectopic tissue. Johan et al. examined infiltrating macrophage phenotype over time in endometriosis lesions in a heterologous mouse model and found that macrophage phenotype was progressively altered over time. Macrophages initially expressed pro-inflammatory markers iNOS and major histocompatibility complex II (MHC II), however at 7 and 14 days post lesion induction a higher proportion of macrophages expressed arginase 1 and CD204 (scavenger receptor A), which are more associated with a tissue remodeling phenotype (136). This study therefore demonstrates that macrophage phenotype in endometriosis lesions is dynamic and progressively changes as lesions develop in the peritoneal cavity. However, as with other studies, the limited number of markers assessed makes it difficult to truly re-capitulate the complex phenotype of macrophages in the tissue.

Endometriosis lesions from women are highly infiltrated by CD68+ macrophages that are present within the stroma of the tissue and can also be found in close proximity to glands (42, 53). Studies in women have strongly implied a role for macrophages in endometriosis, but the mechanistic studies performed in experimental models have significantly improved our understanding of the role of macrophages in the condition. Studies to date have largely focused on defining the role of macrophages in syngeneic mouse models using various cell depletion approaches. A commonly utilized depletion method uses liposomes encapsulating bisphosphonates. These liposomes are taken up by phagocytic cells, which degrade the liposomes, releasing bisphosphonate and causing subsequent cell death. This method therefore selectively depletes phagocytic cells and is non-toxic to non-phagocytic cells, and has been commonly used to deplete phagocytic macrophage populations (137). In a syngeneic mouse model of disease, Bacci et al. used clodronate liposomes and a monoclonal anti-F4/80 antibody to deplete/inhibit peritoneal macrophage function in mice with induced endometriosis and demonstrated that both treatments caused a reduction in growth and blood vessel formation in lesions (53). Adoptive transfer of *in vitro* generated “pro-inflammatory” (stimulated with IFN-γ), “anti-inflammatory” (stimulated with macrophage-colony-stimulating factor and IL-10), or “non-polarized” (stimulated with macrophage-colony-stimulating factor) macrophages lead to differential effects on lesion development. “Non-polarized” macrophages had no effect on lesion number or weight, however adoptive transfer of pro-inflammatory macrophages reduced lesion weight. Conversely, adoptive transfer of anti-inflammatory macrophages caused an increase in lesion weight. The authors noted that lesion architecture was also disrupted in mice which had received adoptive transfer of pro-inflammatory macrophages (53). Together, this data suggests that anti-inflammatory/pro-repair macrophages may be important for the growth and development of lesions and pro-inflammatory macrophages have an antagonistic effect, clearing ectopic endometrial tissue and disrupting lesion architecture. Whilst this data provides an important insight into the roles of macrophage phenotypes in endometriosis, the use of the M1/M2 paradigm is limited and the exact phenotype and phenotypic heterogeneity of macrophages in endometriosis and their role in disease is currently unknown. Capobianco et al. identified Tie-2 expressing macrophages that infiltrated mouse and human lesions. Depletion of Tie-2^+^ macrophages was achieved using a bone marrow chimera from mice expressing a suicide gene (herpes simplex virus type 1 thymidine kinase) expressed under control of the Tie2 promoter into wild-type mice. After treatment with ganciclovir (an anti-viral drug), bone-marrow derived Tie2^+^ cells were selectively depleted and growth of endometriosis lesions was inhibited, with loss of neovascularization and glandular organization in the resultant lesions (127). Sekiguchi et al. demonstrated that VEGFR1 knockout mice had smaller and less vascularized lesions than WT in a mouse model where sections of uterus were sutured onto the peritoneal wall. Using bone marrow chimeras they demonstrated that VEGFR1^+^ cells in lesions were bone marrow derived CD11b^+^ macrophages (125). WT endometriosis mice were also treated with clophosome N which depleted phagocytic cells in the peritoneal cavity at the time of endometriosis induction, and demonstrated that growth and angiogenesis in lesions was reduced (125). A similar study using liposomal bisphosphonate to deplete phagocytic peritoneal populations also demonstrated reduced growth of endometriosis lesions in a rat model (138). Thus, it seems clear that in experimental models of endometriosis, depletion of peritoneal phagocytic macrophage populations inhibits growth and angiogenesis of induced lesions.

Endometriosis lesions exhibit cyclical bleeding in response to ovarian steroids in the same context as the eutopic endometrium, thus lesions can perceived as wounds undergoing recurrent tissue injury and repair (40). The process in lesions has been described to involve epithelial-mesenchymal transition, fibroblast-myofibroblast transdifferentiation, smooth muscle cell metaplasia and fibrosis (139). Macrophages are critical for successful repair and regeneration in tissues; they stimulate local fibroblasts to differentiate into myofibroblasts to facilitate wound contraction (140). During wound repair the proliferation and expansion of local stromal cells is also regulated by macrophages and if the injury is severe, macrophages may activate additional stem cell and local progenitor cell populations that participate in repair (49). In line with these established roles in tissue injury and repair *in vitro* studies have aimed at assessing the interaction between endometrial stromal cells and macrophages in endometriosis. In a co-culture system, culture of endometrial stromal cells with autologous macrophages isolated from women with endometriosis increased the invasive and clonogenic ability of stromal cells (141). Co-culture with ectopic endometrial stromal cells was also shown to decrease the phagocytic capacity of macrophages and increased the survival and proliferation of stromal cells compared to eutopic endometrial stromal cells in a study by Mei et al. (142). A similar effect was also reported by Shao et al. (143). Reciprocal signaling therefore appears to be occurring between ectopic endometrial stromal cells and macrophages, which could contribute to their survival and the formation of endometriosis lesions in the peritoneal cavity, however the precise mechanisms are yet to be elucidated and the specific macrophage populations involved are unknown. Stromal cells derived from ovarian endometrioma were found to express markers of mesenchymal stromal cells (MSCs), formed colony forming units and exhibited multipotency suggesting characteristics of mesenchymal stem-like cells. The MSCs from endometriomas promoted differentiation of monocytes to spindle shaped pro-repair/immunosuppressive macrophages *in vitro* (50). The results suggest that MSC influence macrophages such that they exhibit an immunosuppressive phenotype and support lesion growth. The coordination of monocytes and macrophage activation states during inflammation and repair is tightly and temporally controlled. If disturbances occur at any point in the process this can lead to aberrant repair and the formation of pathological fibrosis (49). For example, persistent activation and sustained recruitment of pro-repair macrophages may contribute to pathological fibrosis (144). Since endometriosis lesions are undergoing consistent and repeated episodes of injury and repair and lesions exhibit fibrotic content, the events required for efficient, scarless repair may be disturbed. Depletion studies have demonstrated that macrophage depletion significantly reduces the fibrosis in lesions. Moreover, adoptive transfer of macrophages polarized *in vitro* to exhibit an M2a phenotype (activated with IL-4) increased the fibrotic content of lesions (139). Thus, it seems that unlike the physiological wound repair process, endometriosis lesions cannot enter the resolution phase of inflammation and repair and the local inflammatory environment causes persistent activation of pro-repair macrophages that contribute to fibrosis.

A role for macrophages in neurogenesis in endometriosis lesions has been established in the literature, suggesting a role in the generation of endometriosis-associated pain. Indeed, nerve infiltration in lesions is positively correlated with higher reported pain scores in women (145). Cholinergic, adrenergic, sensory Aδ and C nerve fibers have been identified in lesions (146, 147), and macrophages are densely populated in areas of high nerve density (55, 148). Greaves et al. reported that in response to estradiol, nerve fibers secreted CCL2 and CSF-1, which attracted macrophages, which in turn secreted neurotrophin 3 and brain-derived neurotrophin factor, stimulating neurogenesis (55). Recently a role for macrophage-derived insulin-like growth factor-1 (IGF-1) as a key signal for nerve outgrowth and sensitization in endometriosis has also been described (54): depletion of peritoneal macrophages by clodronate liposomes reversed abnormal pain behavior in mice with induced endometriosis and notably reduced the number of lesions in the peritoneal cavity, providing a direct link between macrophages and endometriosis-associated pain/lesion development. Macrophages treated with peritoneal fluid from women with endometriosis exhibit an up regulation of IGF-1 at the mRNA level, and mechanistically macrophage-derived IGF-1 increased the growth of embryonic rat dorsal root ganglion explants and this was reversed by an IGF-1 inhibitor. Similarly, IGF-1 inhibition by the IGF-1 receptor inhibitor linsitinib in a mouse model could reverse abnormal pain behaviors (54). Taken together, macrophages are evidently pivotal to facilitating neurogenesis and the generation endometriosis-associated pain symptoms, and this is at least in part mediated by IGF-1. The reciprocal signaling that occurs between macrophages and nerve fibers therefore appears critical in regulating neurogenesis in lesions and neuroinflammation is a key driver of endometriosis pathophysiology.

Studies mechanistically indicate that macrophages play key roles in growth, vascularization, and neurogenesis in lesions, as well as generating pain in the condition and these experiments have given insight into some of the factors expressed by macrophages. However, the phenotype of macrophages in endometriosis has not been fully characterized. Macrophages in endometriosis lesions have long been described as being wound healing and “M2-like,” however few studies have taken into consideration the complexities of macrophage phenotype, where pro-inflammatory and wound-healing like markers often co-exist in response to complex signals from the local tissue microenvironment (76). In humans, lesion resident macrophages express the scavenger receptors CD163 and CD206, associated with hemoglobin scavenging and silent clearance of debris (53). Cominelli et al. also identified CD163^+^ CD206^+^ macrophages in superficial lesions from women, which also expressed high levels of matrix metalloproteinase-27, associated with tissue remodeling (149). Duan et al. characterized nitric oxide synthase (iNOS^+^) pro-inflammatory and CD163^+^ wound healing-like macrophages in mouse endometriosis lesions (139). In a rhesus macaque model of endometriosis, lesions were highly infiltrated by CD163^+^ macrophages (150). Whilst macrophages in endometriosis lesions possessing a “wound healing” like phenotype is synergistic with their role in growth and angiogenesis in lesions, a more comprehensive analysis of macrophage phenotype in endometriosis lesions is required. It is also unknown whether different phenotypes exist within endometriosis lesions, which could play differential roles in pathology, and identifying these populations is key for understanding which macrophage populations are driving pathology.

Identification of endometrial macrophages and bone-marrow monocyte-derived macrophages in endometriosis lesions demonstrates that endometriosis-associated macrophages have different origins, however differential roles for these populations have not yet been investigated. It is possible that the endometrial macrophages in lesions are monocyte-derived since a rapid influx of classical monocytes into the endometrium is observed during endometrial repair. Evidence of embryonically derived tissue resident macrophages in lesions is yet to be demonstrated. Previous studies have demonstrated that depletion of peritoneal macrophages has pronounced effects on lesion size and vascularization (53), depletion of this population translates to reduced number of macrophages in lesions and attenuates pain in mice (54). It remains unknown how endometrial and recruited monocytes contribute to lesion establishment and maintenance. Depletion of different macrophage populations prior to inducing endometriosis in mice and at different time-points during the life-course of the lesion will yield important insights into the role of these pivotal cells in the disorder. Whilst macrophages from 3 origins have been described the true heterogeneity of macrophage phenotype in lesions and in the peritoneal fluid, in endometriosis, is unknown. Application of single cell discovery techniques and digital molecular pathology could provide vital information on the complexities of endometriosis-associated macrophage phenotype, and coupled with *in vivo* functional studies identification of a disease-promoting population that exhibits unique markers that differ from healthy macrophages may be possible.

## The Future: Macrophage Targeted Therapies

Macrophages offer an attractive therapeutic target due to their instrumental role in a number of pathologies (79). Inhibition of macrophage signaling or recruitment, as well as re-education of disease-associated macrophages to a “healthy” phenotype could be of clinical benefit to patients where macrophages are implicated in disease pathophysiology. Identification of disease promoting macrophage populations and a detailed understanding of their regulation, recruitment and phenotype is a fundamental step before the development of therapeutics which specifically target disease-associated macrophages is possible. Due to the pivotal role that macrophages play in many cancers, macrophage-targeted therapies have received much attention in the literature and a number of *in vivo* studies and clinical trials have demonstrated efficacy in using macrophage-mediated treatments to improve clinical outcomes (79). A subset of studies has targeted proliferation of TAMs in an effort to alleviate tumor burden and improve clinical outcomes. Strachan et al. demonstrated that targeting the Csf-1-receptor with a small molecule inhibitor attenuated the turnover rate of TAMs and decreased tumor growth in mouse models of breast and cervical cancer (151). A phase I trial demonstrated a significant reduction in macrophage number in solid tumors after anti-Csf-1r treatment (152), and Csf-1r inhibition showed an improvement in clinical outcomes including improvement of symptoms in patients with diffuse-type giant cell tumors (153). Inhibiting macrophage proliferation therefore appears to be of clinical benefit in cancer models and subsets of cancer patients. Future treatments should aim to specifically target disease-associated macrophage populations; Csf-1 is a key regulator of macrophage proliferation and survival in most tissues and neutralization or inhibition would affect healthy macrophage populations and as such is not an ideal therapy (154). The proliferative capacity of endometriosis lesion-resident macrophages is currently unknown, thus further research is required to determine whether this treatment strategy would be of benefit to women with endometriosis.

Another potential mechanism of therapeutic intervention could involve blocking recruitment of disease-promoting macrophage populations. The CCL2/CCR2 recruitment mechanism is implicated in a number of cancers and a CCR2 inhibitor to be administered alongside chemotherapy is currently in phase 1b trials (155). Inhibition of recruitment may be beneficial in blocking infiltration of macrophages into endometriosis lesions, however the mechanisms, which regulate recruitment into lesions, are currently poorly understood.

Whilst progression of research into macrophage-targeted therapies is promising, current therapies do not specifically target disease-promoting macrophages but have the potential to affect macrophage populations throughout the whole body. However, as our understanding of disease-modified macrophages improves, it is evident that establishing macrophage origins and phenotype heterogeneity in disease are crucial areas of research before specific, targeted treatments can be designed (72). Future work describing macrophage sub-populations, active recruitment mechanisms and macrophage phenotype in endometriosis is therefore critically required before macrophage-targeted treatments may be a possibility for women with endometriosis.

## Author Contributions

CH performed literature search and wrote manuscript. AH provided feedback. EG conceptualized manuscript, provided feedback, and wrote manuscript.

### Conflict of Interest

The authors declare that the research was conducted in the absence of any commercial or financial relationships that could be construed as a potential conflict of interest.
